# Supplementary figures and images for: Development of machine learning models for the prediction of the skin sensitization potential of cosmetic compounds
Source: PeerJ. 2024 Dec 13;12:e18672. doi: 10.7717/peerj.18672 (PMC11648681; doi:10.7717/peerj.18672)

## Sample clustering to detect outliers

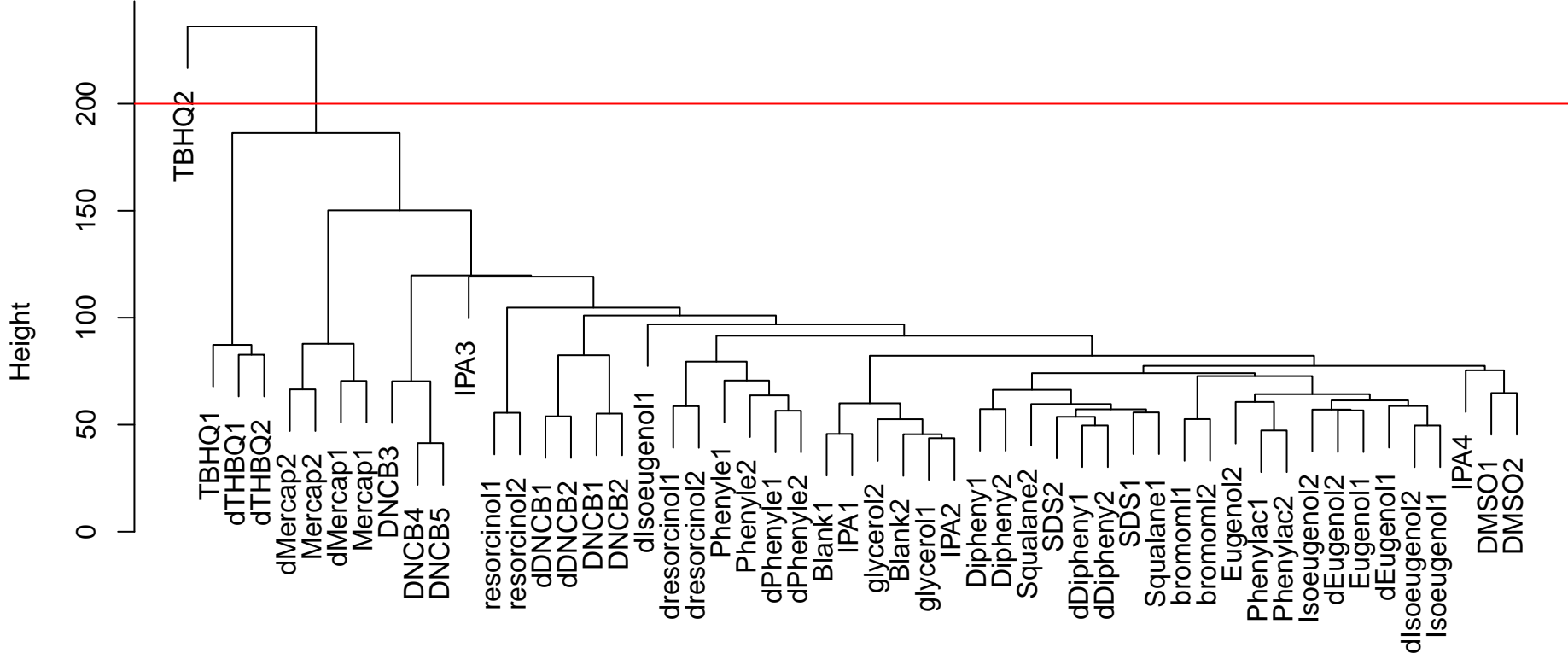

## Sample clustering to detect outliers

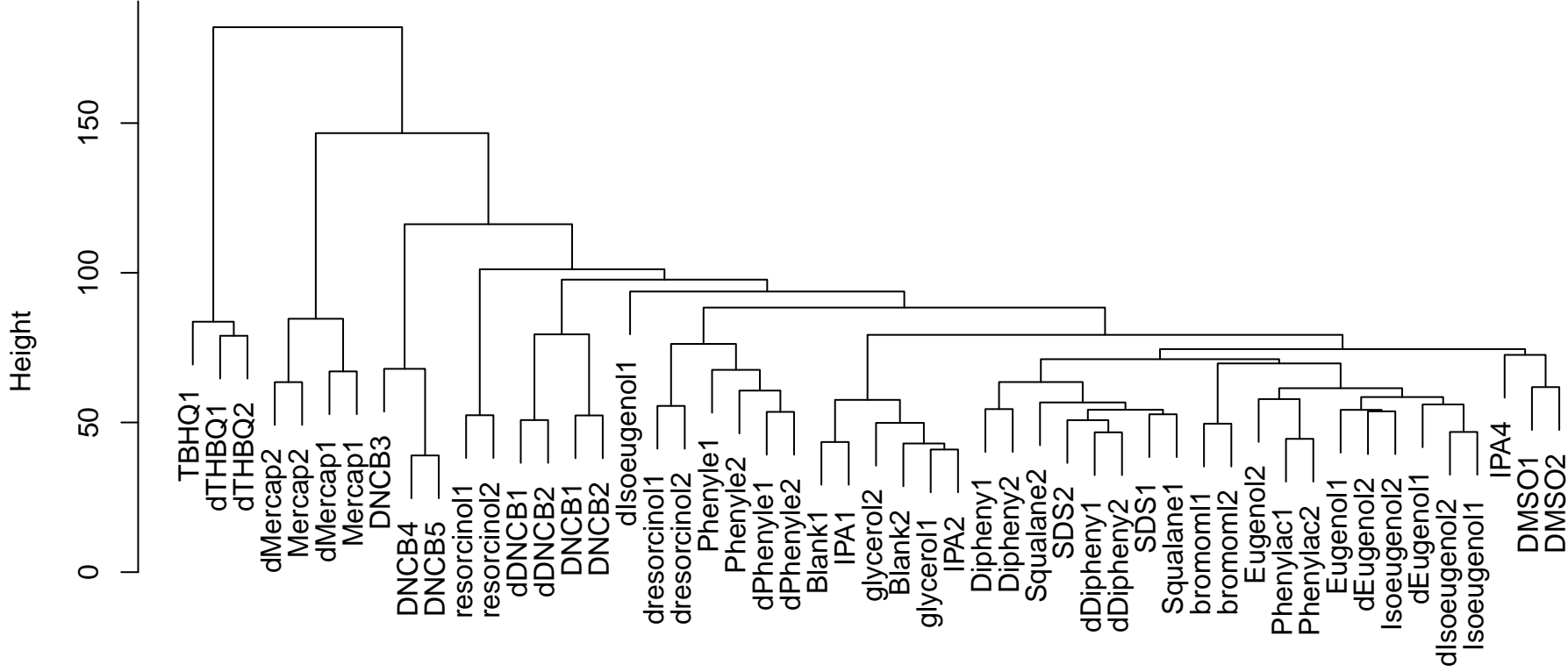

Supplement: Supplemental Information 1 [file peerj-12-18672-s001.pdf]

# Sample dendrogram and trait heatmap

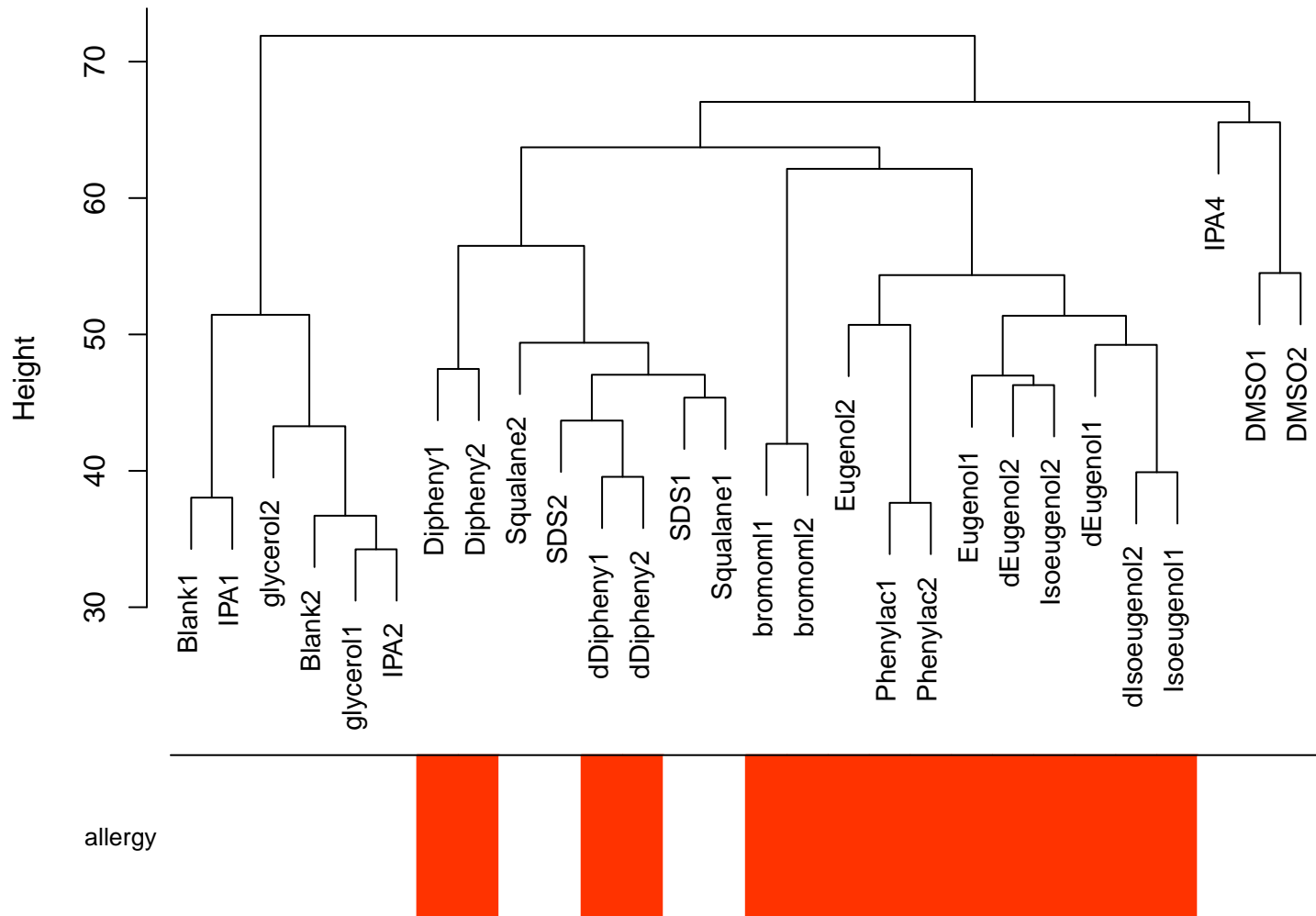

Supplement: Supplemental Information 2 [file peerj-12-18672-s002.pdf]

## Scale independence

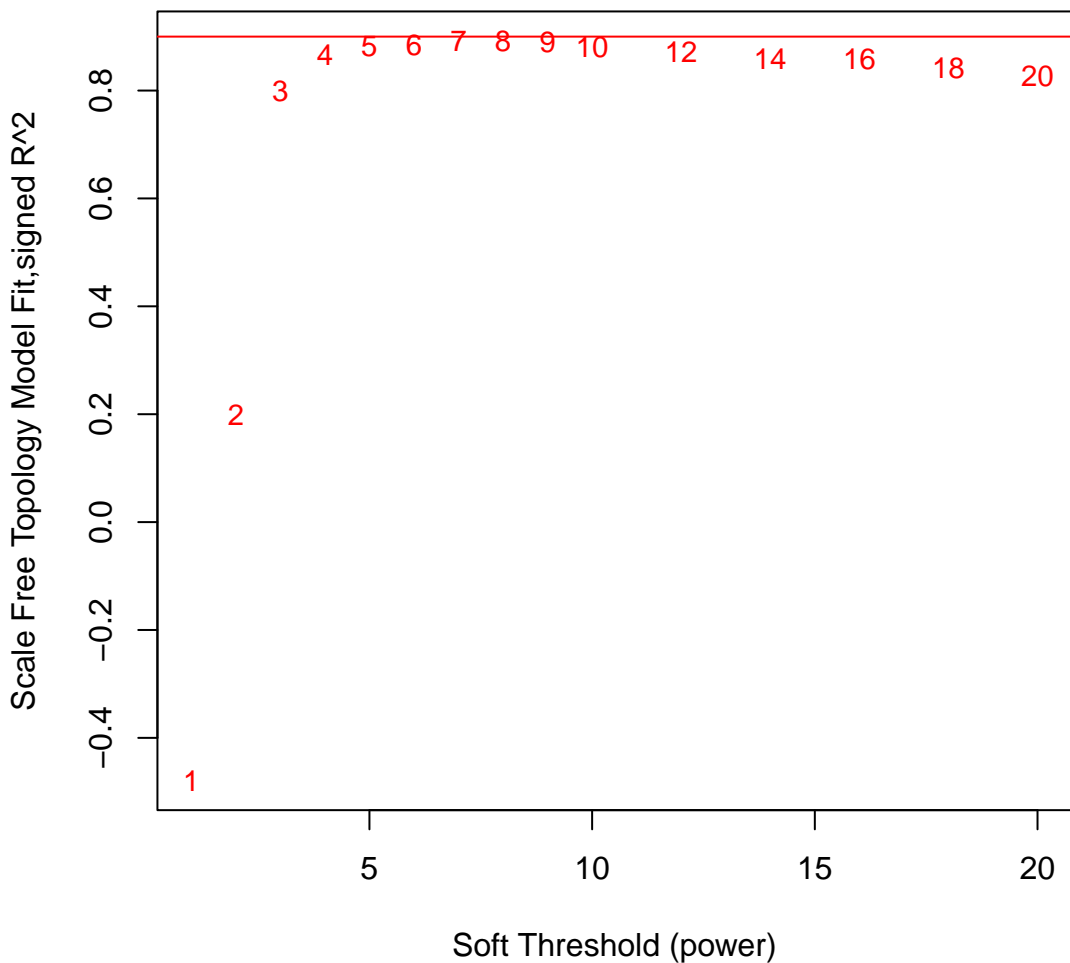

## Mean connectivity

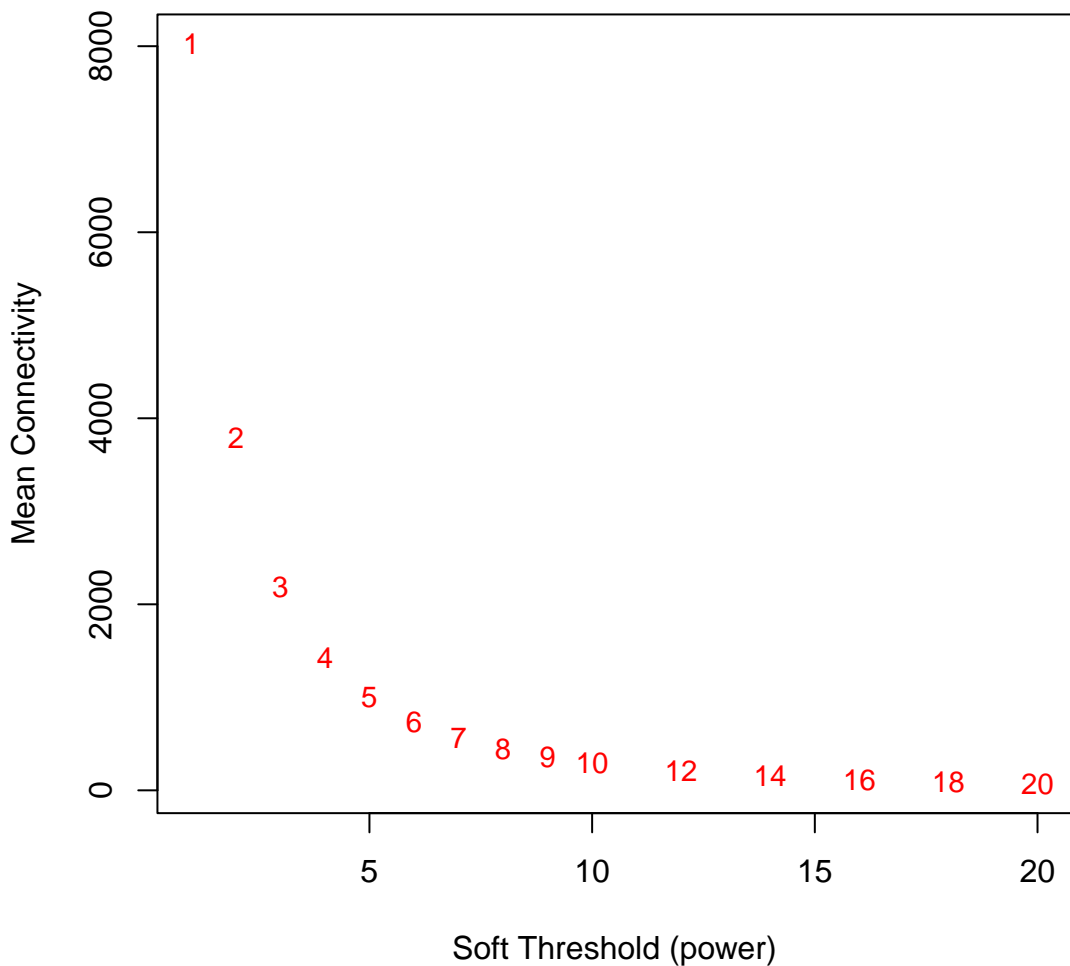

Supplement: Supplemental Information 3 [file peerj-12-18672-s003.pdf]
