# Supplementary material for: Development of machine learning models for the prediction of the skin sensitization potential of cosmetic compounds
Source: PeerJ. 2024 Dec 13;12:e18672. doi: 10.7717/peerj.18672 (PMC11648681; doi:10.7717/peerj.18672)

Table S1 Comparison of Training and Test Accuracies for Vars (50 genes) Methods Using deseq-vst Data Processing


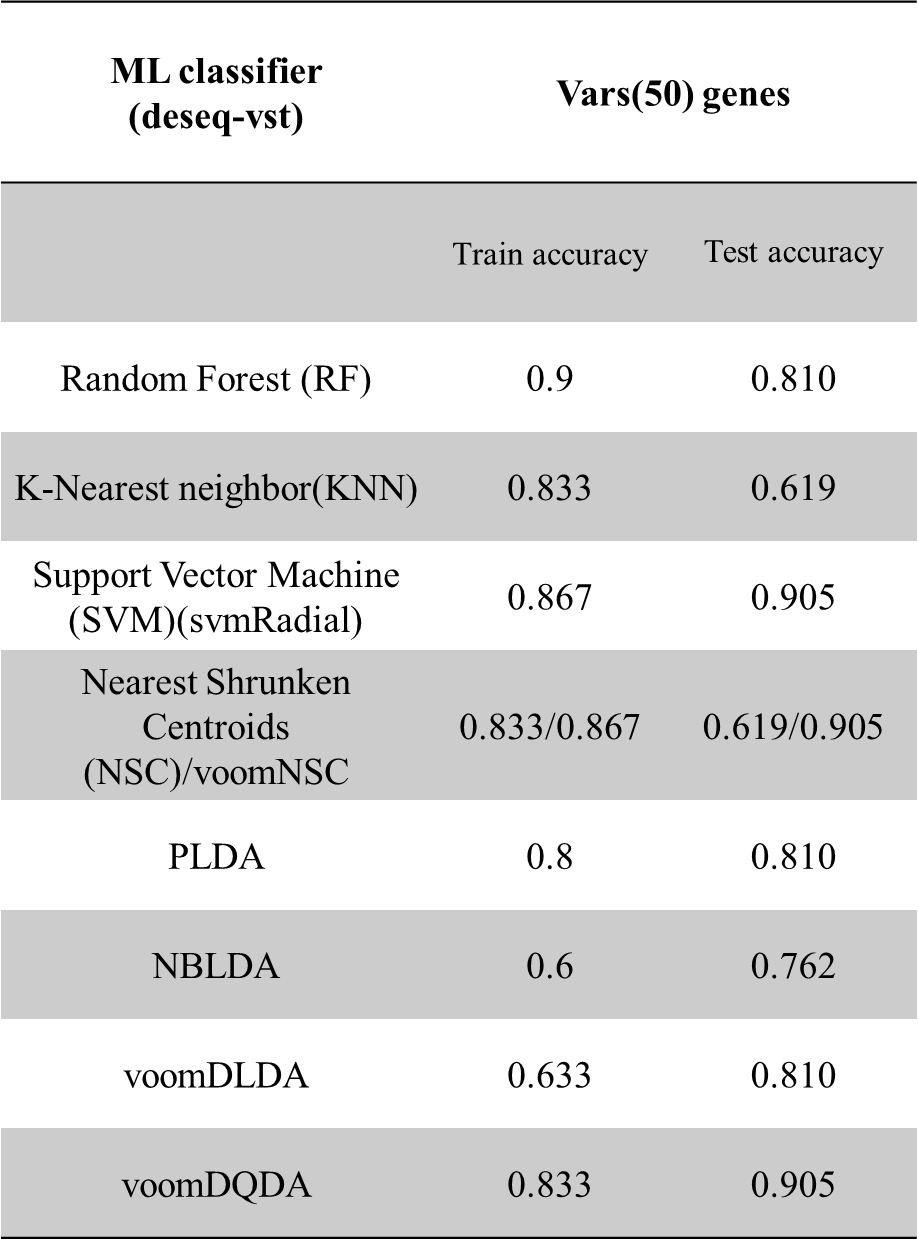

Supplement: Supplemental Information 6 [file peerj-12-18672-s006.docx]
