# Supplementary material for: Development of machine learning models for the prediction of the skin sensitization potential of cosmetic compounds
Source: PeerJ. 2024 Dec 13;12:e18672. doi: 10.7717/peerj.18672 (PMC11648681; doi:10.7717/peerj.18672)

Table S2. Comparison of Training and Test Accuracies for Three Feature Selection Methods Using 60% test data Processed Deseq-vst Data Processing


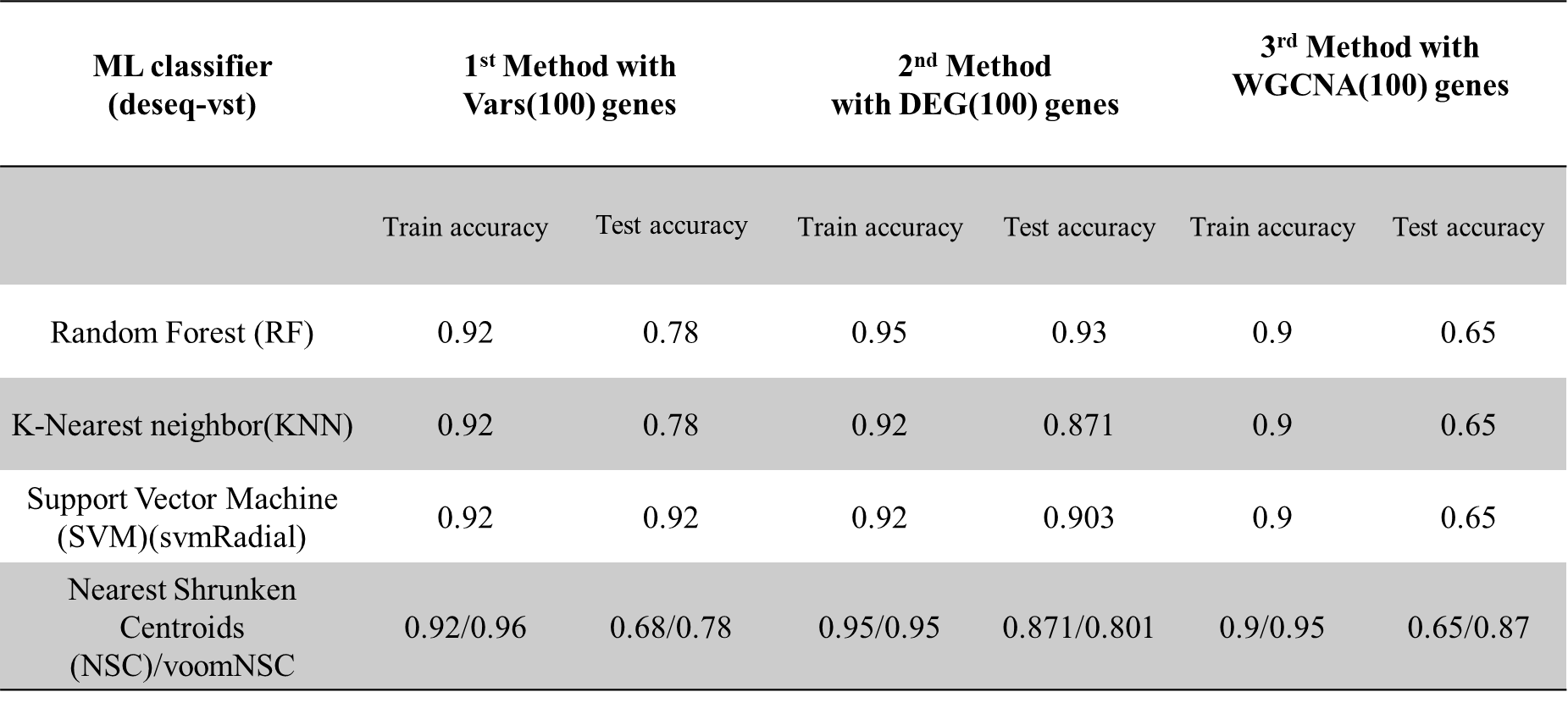

Supplement: Supplemental Information 7 [file peerj-12-18672-s007.docx]
